# Turmeric: A Comprehensive Review of Its Botany, Traditional Uses, Phytochemistry, and Mechanisms as a Functional Food

**DOI:** 10.3390/nu18081197

**Published:** 2026-04-10

**Authors:** Zexuan Wang, Wenhao Zhong, Wenren Zhao, Qian Zhou, Yu Wang, Bing Zhang, Zhijian Lin

**Affiliations:** 1 School of Chinese Materia Medica, Beijing University of Chinese Medicine, Beijing 102488, China; wangzexuan0606@163.com (Z.W.); zhongwenhao233@163.com (W.Z.); zhaowenren22@163.com (W.Z.); 18375069361@163.com (Q.Z.); wangyuxh@163.com (Y.W.); zhangb@bucm.edu.cn (B.Z.); 2 Research Center for Pharmacovigilance and Rational Use of Chinese Medicine, Beijing University of Chinese Medicine, Beijing 102488, China

**Keywords:** turmeric, curcumin, phytochemistry, non-coding RNA, epigenetics, functional food, anticancer, pharmacokinetics

## Abstract

**Objectives:** This review aims to systematically summarize turmeric’s botanical traits, traditional medicinal applications, phytochemical components and their biological activities, and to integrate botanical, phytochemical, molecular and clinical perspectives to provide a comprehensive theoretical foundation and practical guidance for the future scientific research and clinical applications of turmeric as a functional food. **Methods:** A systematic overview and comprehensive analysis were conducted on the existing research about turmeric, covering its botanical characteristics, traditional medicinal application value, the biological mechanisms of major bioactive compounds (especially curcumin), pharmacokinetic properties, and the latest progress in relevant clinical trials. **Results:** Turmeric has important historical and cultural significance in traditional medicine, and its major bioactive compound curcumin is the core of its therapeutic potential, which can modulate antioxidant, anti-inflammatory, and antitumor signaling pathways. Recent studies have found that curcumin exerts significant biological effects by regulating noncoding RNAs (ncRNAs) and epigenetic modifications, showing a promising role in cancer chemoprevention. Meanwhile, curcumin has specific pharmacokinetic properties, and current clinical trials on turmeric and curcumin have made certain progress, yet challenges such as low bioavailability and limited therapeutic efficacy still exist. **Conclusions:** Turmeric, as a widely recognized functional food with rich phytochemicals and diverse biological activities, has great potential in scientific research and clinical application, especially in cancer chemoprevention. Solving the key challenges such as curcumin’s bioavailability and therapeutic efficacy is the core direction for the future development and utilization of turmeric, and the multi-dimensional research perspective can provide more comprehensive support for its practical application as a functional food.

## 1. Introduction

Turmeric (*Curcuma longa*), a perennial herbaceous plant native to South Asia, has a long history as both a culinary spice and a medicinal agent, particularly in Asian cultures. Its rhizomes, which are the source of turmeric powder, have been widely incorporated into traditional diets and herbal remedies across India, China, Southeast Asia, and other regions. The plant thrives predominantly in tropical moist evergreen forestlands spanning the Eastern Himalayas and extends to various subtropical and tropical areas, including parts of the Middle East, Central Asia, and even Western countries, where it has gained popularity [1]. Historically, turmeric has been valued not only for its distinctive flavor and color but also for its purported health benefits, which have been documented in traditional systems such as Ayurveda and Traditional Chinese Medicine. These traditional applications range from anti-inflammatory and digestive uses to wound healing and infection control, underscoring turmeric’s multifunctional role in health maintenance and disease prevention. Given the enormous body of published research spanning thousands of studies, it is not feasible to cite all relevant publications. The references cited in this review were selected based on strict criteria, prioritizing classic and landmark studies reporting initial observations of curcumin’s fundamental properties, key mechanistic investigations into its molecular actions, representative pharmacokinetic/clinical trials evaluating translational potential, and high-impact reviews integrating historical and recent advances.

The primary bioactive constituents of turmeric are curcuminoids, with curcumin being the most abundant and extensively studied. Curcumin is a polyphenolic compound responsible for the characteristic yellow color of turmeric and is credited with a broad spectrum of biological activities. Modern pharmacological research has substantiated many traditional claims, demonstrating curcumin’s potent antioxidant, anti-inflammatory, and antitumor properties [2]. These effects are mediated through the modulation of various molecular targets and signaling pathways, including nuclear factor-κB (NF-κB), cyclooxygenase-2 (COX-2), and caspase cascades, which collectively contribute to its therapeutic potential in chronic inflammatory diseases and cancer [3]. Despite these promising bioactivities, the clinical translation of curcumin faces significant challenges related to its poor aqueous solubility, rapid metabolism, and low systemic bioavailability, which limit its efficacy in vivo [4]. Recent advances have focused on the development of novel delivery systems, such as nanoparticles, liposomes, and polymeric carriers, to enhance curcumin’s stability, bioaccessibility, and targeted delivery, thereby amplifying its therapeutic effects [5,6].

In parallel with the elucidation of curcumin’s biochemical and pharmacological properties, there has been growing interest in understanding its actions at the molecular and epigenetic levels. Emerging evidence highlights the role of curcumin in modulating noncoding RNAs, including microRNAs (*miRNAs*) and long noncoding RNAs (lncRNAs), as well as epigenetic regulators, such as DNA methyltransferases and histone-modifying enzymes [7]. For instance, curcumin has been shown to repress *miR-125b-5p*, a microRNA implicated in oxidative stress regulation, thereby enhancing cellular antioxidant capacity by upregulating catalase and superoxide dismutase activities [8]. Additionally, curcumin influences DNA methylation patterns and histone acetylation status, leading to the reactivation of tumor suppressor genes and suppression of oncogenic pathways, which collectively contribute to its anticancer effects [7]. These epigenetic modifications provide mechanistic insights into curcumin’s pleiotropic actions and position it as a promising multitarget agent for cancer prevention and therapy.

The integration of curcumin’s traditional uses, phytochemical composition, and molecular mechanisms has spurred extensive research into its application as a functional food and therapeutic agent. Notably, the antitumor potential of curcumin has been a focal point, with numerous in vitro and in vivo studies demonstrating its capacity to inhibit tumor cell proliferation, induce apoptosis, and modulate the tumor microenvironment [2,9]. However, the clinical utility of curcumin remains constrained by pharmacokinetic challenges, which have prompted the exploration of nanotechnology-based formulations to improve its delivery and efficacy. Nanoparticles composed of biocompatible materials such as zein, licorice protein isolate, and various polymers have been engineered to encapsulate curcumin, enhancing its solubility, stability, and cellular uptake while preserving or augmenting its bioactivities [3,10,11]. These advanced delivery systems not only potentiate curcumin’s antioxidant and antitumor effects but also reduce systemic toxicity and improve patient outcomes, marking a significant advancement in the functional food and pharmaceutical applications of turmeric-derived compounds.

Despite these promising developments, several knowledge gaps remain, particularly regarding the precise molecular interactions underpinning curcumin’s effects and the translation of preclinical findings into clinical practice. The emerging role of non-coding RNAs and epigenetic regulation offers a novel framework for understanding and harnessing curcumin’s bioactivities, emphasizing the need for further mechanistic studies and well-designed clinical trials. This comprehensive review aims to synthesize current knowledge on turmeric’s botanical characteristics, traditional applications, phytochemical constituents, and molecular mechanisms, with a particular focus on curcumin’s epigenetic roles and antitumor potential. By consolidating these diverse aspects, we seek to provide a theoretical foundation that will guide future research and facilitate the development of turmeric-based functional foods and therapeutic strategies.

## 2. Methods

A systematic literature search was performed to collect relevant publications on *Curcuma longa* (turmeric) and curcumin. Electronic databases including PubMed, Web of Science, and Scopus were used. The search keywords included turmeric, curcumin, phytochemistry, biological activity, anticancer, epigenetics, noncoding RNA, bioavailability, pharmacokinetics, and clinical trial. All English articles published up to 2025 were considered. Studies focusing on the botany, traditional uses, chemical constituents, molecular mechanisms, and clinical applications of turmeric were included. Duplicate, irrelevant, non-English, and abstract-only publications were excluded. Two authors independently screened the literature by title, abstract, and full text to ensure accuracy and eligibility.

## 3. Botanical Characteristics of Turmeric

### 3.1. Classification and Morphological Description

Turmeric (*Curcuma longa*), a prominent member of the Zingiberaceae family, commonly known as the ginger family, comprises numerous species with significant ethnomedicinal and economic value. It is primarily a perennial herbaceous plant characterized by its distinctive yellow rhizomes, which are the underground stems widely utilized for culinary, medicinal, and industrial purposes [12]. Taxonomically, *Curcuma longa* is classified within the genus Curcuma, which includes several species, such as *Curcuma zanthorrhiza* (temulawak), *Curcuma caesia* (blue curcuma), and others that share morphological similarities but differ chemically and geographically [13]. The rhizomes of turmeric are thick, fleshy, and typically exhibit a bright yellow to orange-yellow coloration due to the presence of curcuminoids, a group of polyphenolic compounds responsible for its characteristic color and bioactivity [12]. Morphologically, the plant grows as a clump of leafy stems reaching heights of up to 1 m, with large, oblong leaves and inflorescences that bear pale yellow flowers. The rhizomes are the primary harvested part, consisting of multiple branched segments covered by a brownish skin [14].

**Figure 1:**
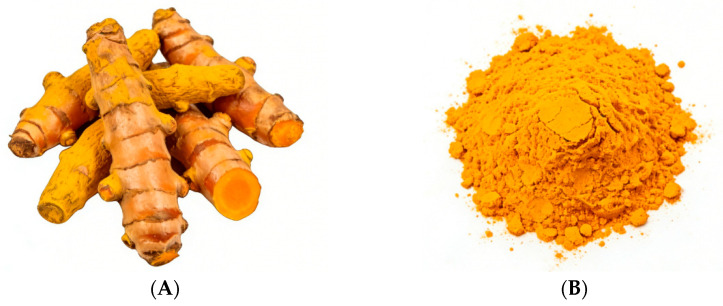
(**A**) fresh rhizomes; (**B**) turmeric powder.

**Figure 2:**
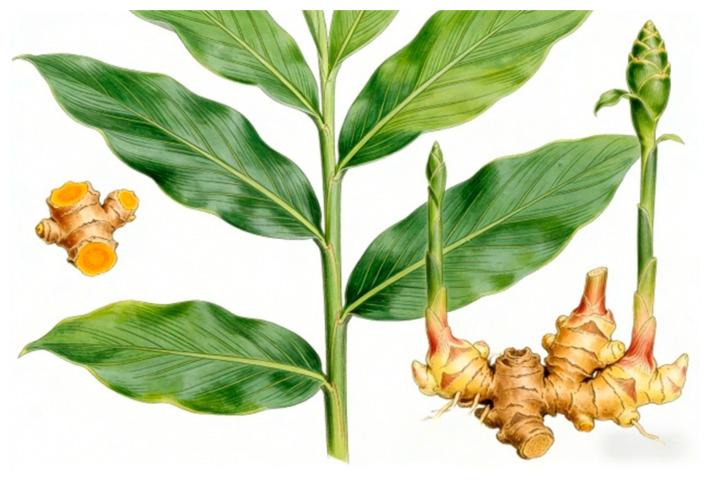
*Curcuma longa* L. (turmeric): whole plant morphology, including leaves, stems, inflorescences, and rhizomes, with an inset of the rhizome cross-section.

Figure 1 and Figure 2 illustrate the macroscopic morphology of turmeric, including the intact plant, fresh rhizomes, and the characteristic deep yellow powder obtained after processing.

The growth environment of turmeric is predominantly tropical and subtropical, and it thrives in warm, humid climates with well-drained, fertile soils rich in organic matter. It is extensively cultivated in South Asia, particularly in India, which is the largest producer and exporter of turmeric globally, as well as in Southeast Asian countries such as Thailand and Indonesia [13,15]. The plant prefers altitudes ranging from sea level to 1500 m and requires a growing period of 7–10 months with adequate rainfall or irrigation. Cultivation conditions, including soil type, climate, and geographical origin, significantly influence the phytochemical composition and metabolite profiles of turmeric rhizomes, leading to variations in curcuminoid content and essential oils [14,15]. This variation necessitates accurate botanical identification and authentication methods to distinguish *Curcuma longa* from closely related species and to ensure quality control in medicinal and functional food applications [13].

Advanced molecular and chemometric techniques have been employed to classify and authenticate turmeric at the species level, addressing challenges posed by morphological similarities and adulteration. Methods such as intron length polymorphism (ILP) markers in genes encoding diketide-CoA synthase and curcumin synthase, Fourier-transform infrared spectroscopy (FTIR) combined with machine learning, and nuclear magnetic resonance (NMR) metabolite fingerprinting have demonstrated high accuracy in differentiating *Curcuma longa* from other Curcuma species and hybrids [13,15]. These approaches facilitate taxonomic classification and help trace the geographical origin and verify the authenticity of turmeric products in the global market [15]. The morphological and taxonomic clarity of turmeric underpins its extensive use in traditional medicine systems, functional foods, and as a source of bioactive compounds, emphasizing the importance of precise botanical characterization for research, quality assurance, and regulatory compliance.

### 3.2. Geographic Distribution and Cultivation

Turmeric (*Curcuma longa*) is predominantly distributed across India and Southeast Asian countries, including Bangladesh, Cambodia, Thailand, China, Malaysia, Indonesia, and the Philippines, with India being the global leader in terms of cultivation area and production. The plant thrives best in humid tropical climates, which provide the warm temperatures and consistent rainfall essential for its growth. For instance, in Kerala, India, a key turmeric-producing region, the climate is characterized by moderate to high humidity and well-distributed rainfall, conditions that are moderately suitable for turmeric cultivation according to the ICAR-National Bureau of Soil Survey and Land Use Planning criteria. The ideal soil for turmeric is well-drained, fertile loam or sandy loam with a pH ranging from slightly acidic to neutral, which facilitates optimal nutrient uptake and rhizome development. However, future climate projections suggest a decline in the area suitable for turmeric cultivation in Kerala by 2050 due to anticipated fluctuations in temperature and increased rainfall, which could adversely affect turmeric productivity. This highlights the sensitivity of turmeric to climatic variables and the need for adaptive cultivation practices to sustain production in its primary growing regions [16]. In addition to climatic suitability, local environmental factors such as soil contamination with heavy metals have been noted in some Southeast Asian regions, which may influence turmeric quality and safety, underscoring the importance of monitoring cultivation sites for environmental pollutants.

Modern cultivation techniques for turmeric are evolving to optimize both yield and the concentration of bioactive compounds, such as curcumin and other curcuminoids, which are responsible for its functional food properties. Recent studies in China, a significant turmeric producer, have emphasized the need for scientific nutrient management and growth cycle alignment to enhance biomass and nutrient accumulation in turmeric plants. Research conducted in Sichuan Province demonstrated that dry matter accumulation in turmeric follows an S-shaped curve, with early growth stages favoring aboveground biomass and later stages prioritizing rhizome and tuber development. Nutrient uptake patterns indicate that potassium and nitrogen are critical macronutrients required in the highest quantities, followed by moderate phosphorus, calcium, and magnesium, and trace micronutrients such as iron, zinc, manganese, and copper. This nutrient dynamic informs stage-specific fertilization strategies that can maximize turmeric growth and potentially increase the concentration of active constituents in the rhizomes [17]. Furthermore, comparative analyses of different Curcuma species cultivated in Thailand revealed significant variability in bioactive compound content, suggesting that both species selection and cultivation conditions influence the phytochemical profile. For example, *Curcuma longa* exhibited the highest curcumin content, while other species showed elevated levels of compounds, such as germacrone, indicating that cultivation practices tailored to specific species can enhance the yield of desired bioactives [18]. Additionally, comprehensive genetic and chemical diversity studies have underscored the importance of molecular and phytochemical standardization in Curcuma species to ensure consistent quality of turmeric products, which is closely tied to cultivation practices and environmental factors. Advances in chloroplast genome sequencing have also provided insights into the phylogenetic relationships within the Zingiberaceae family, offering potential molecular markers that could be used to select and breed turmeric varieties with superior agronomic traits and bioactive profiles [19]. Collectively, these findings highlight that modern cultivation techniques integrating precise nutrient management, species selection, and genetic insights are crucial for optimizing both turmeric yield and its functional food value.

### 3.3. Varieties and Genetic Diversity

Turmeric (*Curcuma longa*) exhibits significant variability among its varieties in terms of active compound content, particularly curcuminoids, which include curcumin, demethoxycurcumin, and bisdemethoxycurcumin. A comprehensive study of 200 Indian turmeric accessions revealed a wide range of variation in rhizome yield traits and curcuminoid content, with curcumin levels ranging from 0.41% to 2.17%, demethoxycurcumin from 0.38% to 1.45%, and bisdemethoxycurcumin from 0.37% to 1.24% [20]. This variability extends to total curcuminoid content, which ranged between 1.26% and 4.55%, indicating substantial genetic diversity within Indian germplasm. Clustering analysis grouped these accessions into seven clusters, with some clusters showing higher mean values for yield and curcuminoid content, suggesting that certain varieties are superior in terms of bioactive compound accumulation. The positive correlation between total curcuminoid content and rhizome traits, such as primary rhizome core diameter and secondary rhizome length, underscores the potential for selecting phenotypic traits as proxies for curcuminoid content in breeding programs. Similarly, phenotypic diversity studies of Indonesian turmeric accessions demonstrated broad variation in morphological traits, including plant height, number of shoots, and rhizome weight, which are linked to the plant’s bioactive potential and agronomic performance. Moreover, different varieties cultivated in diverse geographical locations show distinct essential oil profiles, with major volatile constituents, such as α-phellandrene, 1,8-cineole, α-zingiberene, and turmerones, varying in concentration. This chemical diversity influences not only the pharmacological properties but also the sensory attributes of turmeric. For instance, the Ryudai gold variety contains curcuminoids that exhibit potent anti-inflammatory, wound healing, and anti-diabetic activities, demonstrating that specific cultivars can have enhanced bioactivities linked to their unique phytochemical profiles [21]. In addition, comparative analyses between species, such as *Curcuma amada* and *Curcuma longa*, reveal species-specific volatile organic compounds, further emphasizing the chemical diversity within the genus that affects biological efficacy. Collectively, these findings highlight that the bioactive compound content and associated biological activities vary significantly among turmeric varieties, influenced by genetic makeup and environmental factors, which is crucial for selecting and developing varieties with enhanced functional properties.

Genomic research in turmeric has made significant strides in elucidating the genetic diversity and evolutionary relationships within Curcuma species, providing valuable insights for variety improvement. Molecular marker studies, such as those employing simple sequence repeats (SSR), have revealed high polymorphism and genetic divergence among turmeric accessions, particularly in high-curcumin lines from Northeast India, facilitating the identification of trait-specific genotypes for breeding [22]. These molecular tools enable the assessment of genetic relationships and population structure, which are essential for conserving genetic resources and guiding marker-assisted selection. Furthermore, phylogenomic analyses using targeted low-copy nuclear genes and plastome sequencing across Curcuma species have uncovered ancient hybridization events that have shaped the genus’s evolutionary trajectory, with implications for genetic diversity and speciation [23]. Such hybridization events can introduce novel genetic variation that can be harnessed for crop improvement. Additionally, chloroplast genome studies within the Zingiberaceae family, including Curcuma, have identified hypervariable genes and intergenic regions that serve as molecular markers for phylogeographic and population genetic studies, aiding in the authentication and conservation of turmeric germplasm. Recent advances have also integrated metabolomic profiling with genomic data (geno-metabolomics), as demonstrated in Tenasserim turmeric (*Curcuma candida*), to link phytochemical diversity with genetic lineages and environmental adaptation, providing a comprehensive framework for selecting varieties with desirable metabolic traits. Moreover, machine learning-guided metabolomic fingerprinting has been applied to authenticate turmeric varieties and detect adulteration, ensuring quality control in the functional food and nutraceutical markets [15]. The availability of genomic data has also facilitated the engineering of microorganisms, such as Saccharomyces cerevisiae, to biosynthesize curcumin de novo, highlighting the potential of synthetic biology approaches to complement traditional breeding for enhanced curcumin production. Collectively, these genomic and biotechnological advances provide a robust foundation for turmeric variety improvement by enabling precise selection, enhancing bioactive compound content, and ensuring the sustainability and authenticity of turmeric cultivars in agriculture and industry.

## 4. Traditional Uses of Turmeric

### 4.1. Traditional Medicinal Applications

Turmeric (*Curcuma longa*) has a long history of use in traditional medicinal systems, particularly in Indian Ayurveda and Chinese traditional medicine, where it has been primarily employed for its anti-inflammatory, analgesic, and wound healing properties. In Ayurveda, turmeric is regarded as a potent remedy for a variety of ailments, including digestive disorders, arthritis, skin diseases, and inflammatory conditions. Its rhizome is traditionally used to alleviate inflammation and pain, reflecting its role as a natural anti-inflammatory and analgesic agent. Similarly, in Chinese medicine, turmeric is used to promote blood circulation, resolve blood stasis, and relieve pain, and is often prescribed for conditions involving stagnation and swelling. The therapeutic efficacy in these traditional systems is largely attributed to the presence of curcuminoids, especially curcumin, which exhibits anti-inflammatory, antioxidant, and immunomodulatory effects, as confirmed by modern pharmacological studies [24,25]. In addition to treating internal inflammatory diseases, turmeric has been used topically in traditional formulations to promote wound healing and treat skin infections, leveraging its antimicrobial and anti-inflammatory properties [26]. Traditional preparations of turmeric include powdered rhizomes incorporated into pastes, decoctions, and infusions. For instance, in Ayurveda, turmeric powder is often mixed with water, milk, or oils to create pastes applied to wounds or consumed for systemic effects. In Chinese medicine, turmeric is commonly included in herbal decoctions combined with other botanicals to enhance its therapeutic effects, such as in the Mongolian Xieriga-4 decoction used for urinary system ailments [27]. The rhizomes are typically harvested between 6 and 10 months of age to ensure optimal curcumin content and bioactive volatile oils, which are critical for therapeutic efficacy. Processing methods, such as drying and roasting, also influence the pharmacological properties; light roasting preserves antibacterial activity better than dark roasting, which diminishes curcumin content and antioxidant capacity. Overall, the traditional medicinal applications of turmeric across Asian cultures emphasize its role as a versatile anti-inflammatory, analgesic, and wound healing agent, administered in various forms ranging from topical pastes to oral decoctions, with modern research validating many of these uses through the identification of its bioactive constituents and their mechanisms of action.

### 4.2. Role in Food and Culture

Turmeric (*Curcuma longa*) has held a prominent place as both a culinary spice and a natural dye across various cultures for thousands of years. Its distinctive yellow-orange color and warm, slightly bitter flavor have made it a staple seasoning in many traditional cuisines, particularly in South Asian countries such as India, where it is often referred to as “Haldi” [28]. The application of turmeric as a natural dye extends beyond food; it has historically been used in textiles and cosmetics because of its vibrant pigment, curcumin, which is also responsible for many of its health-promoting properties. In food preparation, turmeric is valued not only for its sensory attributes but also for its preservative and antimicrobial effects, which contribute to food safety and shelf life. For instance, turmeric is incorporated into fermented foods and beverages, such as turmeric-infused kombucha, where it enhances antioxidant and antibacterial activities, thereby improving the functional quality of these products [29]. Moreover, turmeric’s integration into various food matrices, including plant-based milks fortified with turmeric extracts, underscores its expanding role in modern functional foods aimed at delivering health benefits alongside nutrition.

The cultural significance of turmeric is deeply embedded in diverse traditions, where its use transcends culinary applications to include religious rituals, traditional medicine, and social customs. In Ayurveda and other traditional medical systems, turmeric is revered for its therapeutic properties, such as anti-inflammatory and antioxidant effects, which have been harnessed for managing a variety of ailments [24,25]. This medicinal heritage contributes to turmeric’s social and economic value, influencing its cultivation, trade, and consumption patterns worldwide. The spice commands considerable market demand, not only as a food additive but also as a raw material for nutraceuticals, cosmetics, and pharmaceuticals, generating significant socioeconomic impact in producing regions [30]. However, widespread use and high demand have also led to challenges such as adulteration and contamination, necessitating advanced authentication methods, such as loop-mediated isothermal amplification (LAMP) and metabolomic fingerprinting, to ensure product quality and consumer safety [15]. Additionally, the presence of microbial contaminants and mycotoxins in turmeric and other spices underscores the importance of stringent quality control in the spice supply chain.

Culturally, turmeric’s role varies among different societies, reflecting unique culinary traditions and health beliefs. For example, in Middle Eastern and Southeast Asian cuisines, turmeric is a key ingredient in spice blends and rice dishes, whereas in Western countries, turmeric’s popularity has surged owing to growing interest in functional foods and natural remedies [31]. Its socioeconomic importance is also evident in the livelihoods of small-scale farmers and spice traders in developing countries, where traditional cultivation and processing methods persist alongside modern agricultural practices. The integration of turmeric into fermented foods and beverages further illustrates its cultural adaptability and expanding functional role, as these products gain recognition for their probiotic and health-enhancing properties. Collectively, turmeric’s multifaceted applications as a spice, dye, medicinal agent, and functional food component underscore its enduring cultural significance and economic value across global societies.

### 4.3. Modern Scientific Validation of Traditional Efficacy

The traditional applications of turmeric (*Curcuma longa*) have been extensively validated by modern scientific research, bridging ethnomedicinal knowledge with contemporary pharmacology to elucidate the underlying mechanisms of its therapeutic effects. Historically, turmeric has been used in various traditional medicine systems, such as Ayurveda, Unani, and Traditional Chinese Medicine, for ailments ranging from inflammation and wounds to digestive disorders, and more complex diseases, such as cancer and neurodegenerative conditions. Modern studies have confirmed that many of these traditional claims have a solid scientific foundation, primarily attributable to turmeric’s bioactive compounds, especially curcumin and turmeric essential oils.

Pharmacological investigations have demonstrated that curcumin exerts potent anti-inflammatory, antioxidant, anticancer, neuroprotective, hepatoprotective, and immunomodulatory effects by modulating multiple molecular targets and signaling pathways. For instance, curcumin inhibits key inflammatory mediators, such as nuclear factor kappa B (NF-κB), cyclooxygenase-2 (COX-2), tumor necrosis factor-alpha (TNF-α), and various interleukins, consistent with its traditional use in inflammatory and pain-related conditions [32,33]. Furthermore, its antioxidant properties contribute to the protection against oxidative stress-related diseases, which aligns with its traditional applications for wound healing and skin conditions [26].

In cancer research, curcumin and turmeric extracts have shown efficacy in inhibiting tumor cell proliferation, inducing apoptosis, and suppressing metastasis across various cancer types, corroborating traditional claims of anticancer potential. Clinical trials have begun to substantiate these effects, although challenges, such as poor bioavailability, remain [33,34]. Innovative formulations, including nanoparticles, nanogels, and novel bioavailable preparations, are being developed to enhance therapeutic outcomes, reflecting efforts to translate traditional remedies into effective modern treatments.

The neuroprotective effects of turmeric-derived curcumin have been validated in models of Alzheimer’s disease, Parkinson’s disease, and cerebral ischemia, supporting its traditional use for cognitive enhancement and neurological disorders. Curcumin modulates amyloid-beta aggregation, neuroinflammation, and oxidative damage; although its clinical efficacy requires further confirmation [32]. Similarly, anti-arthritic and ant-osteoarthritis effects have been demonstrated through reduction in inflammatory cytokines and modulation of metabolic pathways in chondrocytes, validating its traditional use in rheumatic diseases [35].

Turmeric essential oil, distinct from curcuminoids, also contributes to therapeutic effects with documented anticancer, anti-inflammatory, antimicrobial, and hepatoprotective activities, further supporting the multifaceted traditional uses of the whole plant [36]. Moreover, the integration of turmeric with other herbal extracts, such as in polyherbal formulations, demonstrates synergistic effects, enhancing antibacterial, antioxidant, and anticancer activities, which aligns with traditional multi-herb therapies.

Recent advances in phytochemical standardization, such as validated high-performance liquid chromatography (HPLC) methods, and novel delivery systems, including plant-derived exosomes and mucoadhesive capsules, have improved the quality control, bioavailability, and targeted delivery of turmeric compounds, facilitating the clinical translation of traditional remedies.

In summary, the convergence of traditional knowledge and modern pharmacological research has unveiled the scientific basis for the broad therapeutic spectrum of turmeric. This integration not only validates historical uses but also guides the development of standardized, efficacious, and safe turmeric-based functional foods and phytomedicines. Continued multidisciplinary research, including clinical trials and mechanistic studies, is essential to fully harness the potential of turmeric in contemporary healthcare.

## 5. Phytochemical Components of Turmeric

### 5.1. Major Active Constituents: Curcumin and Its Derivatives

Curcumin, the principal bioactive polyphenolic compound in turmeric (*Curcuma longa*), is a diarylheptanoid characterized by a distinctive diketone structure and two phenolic rings connected by a seven-carbon linker, which exhibit α,β-unsaturated carbonyl groups. Its primary derivatives include demethoxycurcumin (DMC) and bisdemethoxycurcumin (BDMC), which structurally differ by the absence of one or both methoxy groups on the aromatic rings, respectively. These structural variations influence their physicochemical properties and biological activities. Typically, turmeric rhizomes contain curcumin at approximately 60–70%, with DMC and BDMC comprising approximately 20–25% and 10–15%, respectively, although these proportions can vary depending on the plant source, processing, and extraction methods. The presence of these curcuminoids collectively contributes to turmeric’s therapeutic effects, including antioxidant, anti-inflammatory, and anticancer activities. Notably, BDMC has been reported to exhibit stronger antibacterial photodynamic activity than curcumin itself, indicating that subtle structural differences modulate bioactivity profiles [37]. Furthermore, curcumin and its analogs possess critical thiol-reactive α,β-unsaturated carbonyl groups that are pivotal for their biological functions, including modulation of cell death pathways and proteasome inhibition in cancer cells. The chemical diversity within curcuminoids thus underpins a spectrum of pharmacological potentials, making the understanding of their distinct structures and relative abundances essential for optimizing therapeutic applications.

The efficiency of extracting curcumin and its derivatives from turmeric is significantly influenced by the extraction methodology employed, which affects the yield, purity, and stability of the bioactive compounds. Conventional solvent extraction methods using ethanol or acetone are widely used but often suffer from low solubility of curcuminoids and degradation during processing. Recent advances have introduced innovative techniques, such as nano-formulation, supercritical fluid extraction, and encapsulation strategies, to enhance extraction efficiency and bioavailability. For example, the raw-to-nano strategy enables the direct formulation of turmeric nanoparticles from raw turmeric, leveraging inherent biopolymers to encapsulate and protect curcumin, thereby improving gastrointestinal stability and bio-accessibility compared to curcumin crystals [38]. Similarly, smartFilm technology enhances dermal and transdermal delivery of curcumin by increasing particle dispersion and creating a local aqueous meniscus that facilitates skin penetration. Extraction methods that preserve the chemical integrity of curcumin and its analogs are crucial, as curcumin is prone to rapid metabolism and degradation, which limits its therapeutic efficacy. Moreover, the incorporation of curcumin into delivery systems, such as nanostructured lipid carriers, hydrogel beads, or protein-based nanoparticles, has been shown to improve stability and controlled release, indirectly reflecting on extraction and formulation efficiency. Extraction efficiency is also affected by the maturity of the turmeric plant material, as demonstrated in studies on turmeric-derived exosome-like nanoparticles, where the bioactive compound content and delivery performance vary with plant maturity. Overall, optimizing extraction methodologies to maximize yield and preserve the functional integrity of curcumin and its derivatives is a critical step in harnessing their full potential as functional food ingredients and therapeutic agents.

### 5.2. Other Secondary Metabolites

Turmeric (*Curcuma longa*) is renowned not only for its curcuminoids but also for a diverse array of other secondary metabolites, including volatile oils (essential oils), polyphenols, and flavonoids, which contribute significantly to its pharmacological and functional food properties. These compounds have been extensively studied for their bioactivities, which complement and, in some cases, potentiate the effects of curcuminoids; thus, highlighting the importance of considering the entire phytochemical profile in turmeric’s therapeutic applications.

Volatile oils in turmeric primarily consist of sesquiterpenes and monoterpenes, such as turmerones, ar-turmerone, α-thujene, and β-elemene, which are differentially synthesized during plant development and vary among cultivars and environmental conditions. For instance, essential oil content and composition fluctuate with leaf age, with 6-month-old turmeric leaves showing the highest expression of genes involved in essential oil biosynthesis, correlating with increased yields of monoterpenes and sesquiterpenes. These volatile oils exhibit antimicrobial, anti-inflammatory, and antioxidant properties, contributing to turmeric’s broad spectrum of biological activities. Studies on black turmeric (*Curcuma caesia*) have also revealed significant variations in phenolics, flavonoids, and alkaloids, with positive correlations among these metabolites suggesting coordinated biosynthesis and potential pharmacological synergy. Moreover, volatile oleoresins and curcuminoids have been identified in turmeric-derived exosome-like nanoparticles (TELNs), indicating their natural packaging and potential for enhanced bioavailability and biological effects.

Polyphenols and flavonoids in turmeric include compounds, such as kaempferol derivatives, quercetin, rutin, and apigenin, which are well-known for their antioxidant and anti-inflammatory activities. Hydro-alcoholic extracts of turmeric combined with other herbs demonstrate significant antioxidative properties and protective efficacy against toxins, such as aflatoxin B1, largely attributed to their phenolic content and free radical-scavenging abilities. Additionally, polyphenols isolated from Costa Rican turmeric rhizomes have shown strong antioxidant activity and varied curcuminoid content, with regional differences influencing their phytochemical profiles and bioactivities. Such polyphenols contribute to the overall health benefits of turmeric, including modulation of oxidative stress and inflammation, which are central to many chronic diseases.

The bioactivities of these secondary metabolites extend to immunomodulatory, anticancer, neuroprotective, and antimicrobial effects. For example, the volatile oils and polyphenols in turmeric have demonstrated antifungal activity against pathogens affecting turmeric, such as *Colletotrichum gloeosporioides*, indicating their role in plant defense and potential for biocontrol applications. Furthermore, flavonoids and phenolic compounds in turmeric exhibit synergistic antioxidant effects, enhancing cellular protection against oxidative damage and contributing to anti-aging and neuroprotective mechanisms. In cancer models, the combination of curcumin with other turmeric metabolites or external agents shows enhanced antiproliferative effects, suggesting that the complex mixture of secondary metabolites may act synergistically to modulate multiple signaling pathways.

The potential synergistic roles of these components are increasingly being recognized. Studies have indicated that extracts containing polysaccharides, curcuminoids, and terpenoids have differential effects on inflammatory and bone resorptive diseases; complex extracts sometimes outperform isolated curcuminoids, likely due to additive or synergistic interactions among secondary metabolites. Similarly, fermented turmeric products enriched in flavonoids and curcuminoids show improved biological activities, including anti-inflammatory and antioxidant effects, supporting the concept that the whole phytochemical matrix enhances efficacy. The presence of flavonoids and phenolics alongside curcuminoids may improve stability, bioavailability, and target multiple molecular pathways, thus providing a multifaceted therapeutic approach.

In summary, volatile oils, polyphenols, and flavonoids in turmeric contribute substantially to its bioactivity profile beyond curcuminoids alone. Their diverse chemical nature and biological functions suggest that they play critical roles in plant defense and therapeutic potential. Importantly, these secondary metabolites likely act in concert, producing synergistic effects that enhance turmeric’s efficacy as a functional food and medicinal agent. Future research focusing on integrated phytochemical interactions and optimized formulations considering these metabolites will be crucial for advancing turmeric applications in health and disease management.

### 5.3. Stability and Bioavailability of Components

Curcumin, the principal bioactive polyphenol in turmeric, has notable therapeutic properties, including anti-cancer, anti-inflammatory, antioxidant, and antimicrobial effects. However, its clinical and functional food applications are severely limited by its intrinsic chemical instability and poor bioavailability. The chemical stability of curcumin is highly sensitive to environmental factors, such as light, pH, and temperature. Studies have demonstrated that curcumin undergoes photodegradation under visible and solar light, primarily through hydrolytic and oxidative fragmentation of its heptadienedione moiety, with optimal stability only at neutral pH and low light intensity. The presence of minor curcuminoids can confer some protection under low irradiation; however, curcumin is generally prone to degradation, which raises questions about the biological relevance of non-enzymatic degradation pathways in its bioactivity [39]. Furthermore, pulsed light treatment has been shown to induce photochemical transformations, such as dimerization, which may paradoxically enhance intracellular antioxidant capacity despite reducing chemical antioxidant measures. The intrinsic electronic structure of curcumin contributes to its photostability and photodynamic therapeutic potential, with fragmentation being an unlikely key route to biological activity.

In vivo, curcumin is rapidly metabolized and transformed, further complicating its bioavailability profile. Its poor water solubility and rapid systemic clearance result in low plasma and tissue levels, thereby limiting its therapeutic efficacy [40]. Bioavailability is influenced not only by the physicochemical properties of curcumin but also by the nature of the delivery system and environmental factors within the gastrointestinal tract. Therefore, improving the stability and bioavailability of curcumin is critical for effective clinical and functional food applications.

Recent advances have focused on nanoformulations and delivery systems that enhance curcumin solubility, protect it from degradation, and improve its absorption and sustained release. Nanocarriers, such as nanoparticles, liposomes, micelles, nanoemulsions, and solid lipid nanoparticles, have been extensively studied. For example, amphiphilic derivatives of chitosan can stabilize curcumin molecules in their keto form, significantly reducing degradation in aqueous environments. Zein hydrolysate-based nanocomplexes encapsulate curcumin within hydrophobic cores, enhancing water solubility, encapsulation efficiency (>70%), and providing sustained release over 91 h [41]. Similarly, soy protein-encapsulated curcumin nanoparticles prepared via pH-shift methods exhibit high encapsulation efficiency (up to 97.43%) and improved thermal and photostability. Pickering emulsions stabilized by protein-polysaccharide complexes, such as glycosylated whey protein isolate, have demonstrated high encapsulation efficiency (~96.64%) and enhanced bio-accessibility (~40.34%) of curcumin [42]. Other polysaccharide-based systems, including chitosan/gum arabic nanoparticles and gum arabic-stabilized emulsions, have also been effective in improving curcumin stability and release profiles.

Metal oxide aerogels loaded with curcumin have been shown to stabilize the organic moiety via curcumin-network interactions; however, the nature of the metal oxide influences the degree of quenching and complexation. Nanostructured lipid carriers using conjugated linoleic acid as a liquid lipid synergistically enhanced the antioxidant activity and bio-accessibility of curcumin (up to 85.7%). Furthermore, cocrystallization with L-carnitine significantly improves dissolution rates and oral bioavailability, with pharmacokinetic studies showing a 6.3-fold increase in AUC and a 10.7-fold increase in Cmax compared to pure curcumin [43].

Bioavailability enhancement strategies also include the use of hydrophobin-based nanoparticles that improve water dispersibility and stability, resulting in facilitated curcumin release and increased cytotoxicity against cancer cells. Nanocrystals stabilized by phospholipids with polyethylene glycol have demonstrated colloidal stability, low macrophage clearance, and anticancer activity comparable to that of commercial cytostatics. Nanoemulsions stabilized with modified phosphatidylcholine significantly reduce tumorigenesis in vivo, highlighting the therapeutic potential of bioavailable curcumin formulations. Surfactin-based nanoencapsulation improves curcumin bioavailability and sustained release, with enhanced antioxidant effects in cellular models [44]. Additionally, zein/sophorolipid nanoparticles prepared via one-step self-assembly improve aqueous solubility by approximately 246-fold and increase bioaccessibility sixfold.

The encapsulation of curcumin in protein-polysaccharide composite systems, such as lotus seed protein-pectin microcapsules, enhances solubility, photostability, thermostability, and provides sustained release during gastrointestinal digestion. Similarly, shellac-gum arabic-Tween 20 nanodispersions improve acid stability and bioaccessibility, with antioxidant activity superior to free curcumin. Pickering emulsions formulated with soybean protein isolate and chitosan complexes show enhanced bioaccessibility (up to 51%) and extended stability, making them promising for elderly nutrition applications. Moreover, cosdelivery systems combining curcumin with other bioactives or oils have demonstrated synergistic effects and improved functional outcomes.

In summary, curcumin exhibits remarkable stability and promising biological activities, yet its clinical translation is severely hindered by three core limitations: poor aqueous solubility, rapid metabolic degradation, and low systemic bioavailability [39,40,45,46]. To overcome these challenges, numerous nanotechnology-based delivery systems—including liposomes, polymeric nanoparticles, micelles, and solid lipid carriers—have been developed. These formulations effectively enhance curcumin’s solubility, protect it from premature metabolism, improve cellular uptake, and enable targeted tissue delivery, thereby significantly increasing its bioavailability and unlocking its full therapeutic potential in cancer treatment and other clinical applications [38,42,44].

## 6. Molecular Mechanisms of Curcumin and Its Anticancer Potential

**Figure 3:**
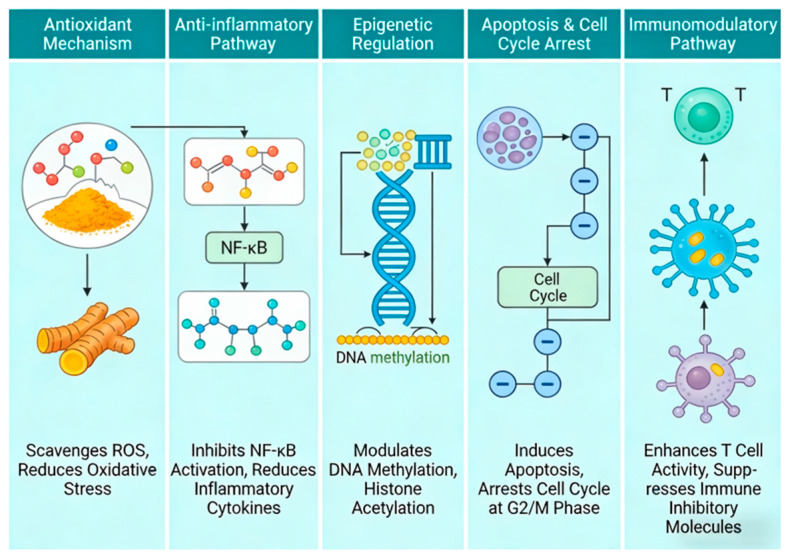
Schematic overview of curcumin’s anticancer mechanisms.

Curcumin, the primary bioactive constituent of turmeric, exerts its multifaceted biological and anticancer effects through a complex network of signaling pathways and regulatory mechanisms. To provide a clear and intuitive overview of these core mechanisms, a schematic diagram is presented in Figure 3, which summarizes the five key pathways underlying curcumin’s activity: antioxidant defense, anti-inflammatory signaling, epigenetic modulation, induction of apoptosis and cell cycle arrest, and immunoregulation. The following sections will elaborate on each of these pathways in detail, highlighting the molecular targets and functional consequences of curcumin’s actions.

### 6.1. Antioxidant and Anti-Inflammatory Mechanisms

Curcumin, the principal bioactive compound of turmeric (*Curcuma longa*), exerts potent antioxidant and anti-inflammatory effects through complex molecular pathways that regulate oxidative stress and the expression of inflammatory cytokines. At the molecular level, curcumin modulates key signaling pathways, such as nuclear factor kappa B (NF-κB) and nuclear factor erythroid 2-related factor 2 (Nrf2), which are pivotal in controlling inflammatory responses and cellular antioxidant defenses, respectively. Curcumin inhibits the activation of NF-κB, a transcription factor that regulates the expression of pro-inflammatory cytokines, including tumor necrosis factor-alpha (TNF-α), interleukin-6 (IL-6), and interleukin-1 beta (IL-1β). This suppression leads to reduced production of these cytokines, thereby attenuating inflammation in various pathological conditions, such as rheumatoid arthritis, cardiovascular diseases, and chronic kidney disease [47,48]. Concurrently, curcumin activates the Nrf2 pathway, which induces the expression of antioxidant enzymes, including heme oxygenase-1 (HO-1), superoxide dismutase (SOD), catalase, and glutathione peroxidase (GPx). This activation enhances cellular antioxidant capacity by scavenging reactive oxygen species (ROS) and mitigating oxidative damage. The dual regulation of NF-κB and Nrf2 pathways by curcumin establishes a balance between pro-inflammatory and antioxidant responses, which is crucial for maintaining cellular homeostasis under oxidative stress conditions.

In vitro and in vivo studies have demonstrated that curcumin reduces oxidative stress markers, such as malondialdehyde (MDA), and enhances total antioxidant capacity (TAC) and superoxide dismutase (SOD) activity, indicating its efficacy in neutralizing free radicals and protecting tissues from oxidative injury [48]. For example, in models of ethanol-induced hepatic toxicity, curcumin pretreatment upregulated antioxidant enzymes and downregulated inflammatory mediators, including IL-1β, IL-6, and TNF-α, highlighting its hepatoprotective role through modulation of oxidative stress and inflammation. Similarly, in cardiovascular and renal diseases, curcumin supplementation has been shown to reduce oxidative stress and inflammatory markers, contributing to improved clinical outcomes [49].

Curcumin suppresses the MAPK signaling pathway and inhibits cyclooxygenase-2 (COX-2) and inducible nitric oxide synthase (iNOS), further reducing the production of inflammatory mediators. Additionally, curcumin modulates apoptotic pathways by regulating pro- and anti-apoptotic proteins, such as Bax and Bcl-2, contributing to its protective effects against inflammation-induced cell death. The compound inhibits NF-κB activation, which is associated with the downregulation of matrix metalloproteinases (MMPs) involved in tissue remodeling and inflammatory damage.

Recent advances have highlighted the enhanced bioavailability and efficacy of curcumin through novel formulations, such as nanoparticles and liposomal encapsulation, which improve its stability and cellular uptake, thereby potentiating its antioxidant and anti-inflammatory actions. For instance, nanoencapsulated curcumin exhibits superior inhibition of inflammatory responses and oxidative stress compared to free curcumin, as demonstrated in cutaneous inflammation and ischemic stroke models.

Moreover, curcumin’s antioxidant and anti-inflammatory mechanisms are implicated in its therapeutic potential across diverse clinical conditions, including neurodegenerative diseases, diabetes, cancer, and inflammatory bowel diseases. It modulates immune responses by restoring redox balance and suppressing pro-inflammatory cytokines, which contributes to its broad-spectrum efficacy. Curcumin also influences epigenetic regulators, such as microRNAs and long noncoding RNAs, which play roles in inflammatory and oxidative stress pathways, further elucidating its molecular mechanisms.

In summary, curcumin exerts antioxidant and anti-inflammatory effects primarily by inhibiting NF-κB-mediated expression of inflammatory cytokines and activating the Nrf2 antioxidant pathway. These molecular actions result in decreased oxidative stress, reduced inflammation, and protection against cellular damage. The modulation of additional signaling cascades and apoptotic regulators further enhances its therapeutic potential. Novel delivery systems that improve curcumin bioavailability are promising strategies to maximize these beneficial effects, supporting its role as a functional food component and therapeutic agent in managing oxidative stress and inflammatory disorders.

### 6.2. Epigenetic Mechanisms Regulated by Non-Coding RNAs

The epigenetic regulation mediated by non-coding RNAs (ncRNAs), including microRNAs (*miRNAs*) and long non-coding RNAs (lncRNAs), represents a critical mechanism through which curcumin, the principal bioactive compound of turmeric, exerts its multifaceted biological effects, especially in cancer prevention and therapy. Curcumin modulates the expression profiles of various ncRNAs, thereby influencing gene expression without altering the underlying DNA sequence. Specifically, curcumin has been shown to regulate *miRNAs* and lncRNAs that are pivotal in controlling cellular processes, such as proliferation, apoptosis, migration, and metastasis in cancer cells. For instance, curcumin downregulates oncogenic lncRNA *H19* and modulates the expression of *miRNAs* involved in tumor suppression, contributing to the inhibition of malignant phenotypes in hepatocellular carcinoma by silencing the Wnt/β-catenin signaling pathway through epigenetic downregulation of enhancer of zeste homolog 2 (*EZH2*) [7,50]. Furthermore, in colorectal cancer, curcumin suppresses tumor metastasis by regulating ncRNAs that influence the expression of heparanase (*HPSE*), a key enzyme in extracellular matrix degradation and tumor microenvironment remodeling, thus inhibiting the IL-6/STAT5 signaling axis. The modulation of ncRNAs by curcumin extends beyond cancer, as it also affects lncRNA expression related to metabolic disorders such as obesity, where curcumin regulates lncRNA *SNHG9* to improve insulin sensitivity and reduce inflammation. These findings underscore the ability of curcumin to orchestrate complex epigenetic networks through ncRNA regulation, highlighting its potential as a multi-target epigenetic modulator.

Non-coding RNAs play essential roles in the regulation of cancer cell proliferation, apoptosis, and metastasis by acting as fine-tuners of gene expression at transcriptional and post-transcriptional levels. *miRNAs*, typically 22 nucleotides in length, regulate gene expression by binding to the 3′-untranslated regions (3′-UTRs) of target mRNAs, leading to mRNA degradation or translational repression. Dysregulation of *miRNAs* is frequently observed in cancer, where oncogenic miRNAs (oncomiRs) promote tumor growth and metastasis, whereas tumor suppressor miRNAs inhibit these processes. Curcumin and other polyphenols have been shown to restore the balance of miRNA expression by downregulating oncomiRs and upregulating tumor suppressor miRNAs, thereby inhibiting cancer progression. Similarly, lncRNAs, which are transcripts longer than 200 nucleotides without protein-coding potential, modulate gene expression through diverse mechanisms, including chromatin remodeling, transcriptional interference, and acting as miRNA sponges. The aberrant expression of lncRNAs is implicated in tumorigenesis and metastasis. Curcumin can downregulate oncogenic lncRNAs, such as *H19*, and modulate others involved in epithelial–mesenchymal transition (EMT) and tumor microenvironment remodeling, further exemplifying its epigenetic regulatory capacity [50]. This intricate regulation by ncRNAs influences key signaling pathways, such as NF-κB, PI3K/Akt, and Wnt/β-catenin, which are central to cancer cell survival and dissemination.

The interaction network between ncRNAs and protein targets forms a complex regulatory system that is crucial for maintaining cellular homeostasis and is often disrupted in cancer. Curcumin modulates this network via epigenetic modifications that affect the expression and activity of DNA methyltransferases (DNMTs), histone deacetylases (HDACs), and other chromatin-modifying enzymes, which in turn influence ncRNA expression and function [7]. For instance, curcumin inhibits DNMTs, leading to DNA demethylation and reactivation of silenced tumor suppressor genes, while also modulating histone acetylation and methylation to restore normal chromatin accessibility. These epigenetic changes are coupled with alterations in ncRNA profiles, creating a feedback loop that reinforces anti-tumor gene expression patterns. Moreover, ncRNAs can regulate epigenetic modifiers, adding another layer of complexity to the regulatory network. This bidirectional interaction is significant in chemoprevention, as targeting ncRNAs with phytochemicals, such as curcumin, can disrupt oncogenic signaling and reprogram cancer cells towards apoptosis and growth arrest.

In summary, epigenetic mechanisms mediated by ncRNAs represent a vital axis through which curcumin exerts its chemopreventive and therapeutic effects. By modulating miRNA and lncRNA expression, curcumin influences cancer cell proliferation, apoptosis, invasion, and metastasis. The complex interplay between ncRNAs and protein targets, modulated by curcumin’s epigenetic actions, underscores the potential of this phytochemical as a multi-target agent in cancer prevention and treatment. Continued research into these ncRNA-mediated networks will facilitate the development of novel epigenetic therapies harnessing the power of natural compounds, such as curcumin.

### 6.3. Apoptosis and Cell Cycle Regulation

Curcumin, the principal bioactive polyphenol derived from turmeric (*Curcuma longa*), exerts potent anticancer effects by inducing apoptosis and modulating cell cycle progression in various cancer cell types. The molecular mechanisms underlying curcumin-induced apoptosis involve both the intrinsic mitochondrial pathway and the extrinsic death receptor pathway. In the mitochondrial pathway, curcumin triggers the loss of mitochondrial membrane potential, leading to the release of cytochrome c and the subsequent activation of caspase-9 and caspase-3, culminating in programmed cell death. This has been supported by evidence showing that curcumin treatment increases pro-apoptotic proteins, such as Bax, and decreases anti-apoptotic proteins, such as Bcl-2, thereby shifting the balance toward apoptosis in cancer cells [51]. Additionally, curcumin downregulates key survival signaling pathways, including the PI3K/AKT/GSK-3β axis, which further facilitates mitochondrial apoptosis [51]. The extrinsic apoptosis pathway is also modulated by curcumin, as demonstrated by the upregulation of death receptors and activation of caspase-8, which can amplify apoptotic signaling. Furthermore, curcumin and its analogs have been shown to induce apoptosis via reactive oxygen species (ROS) generation, which contributes to mitochondrial damage and the activation of apoptotic cascades.

In parallel with apoptosis induction, curcumin exerts significant effects on cell cycle regulation, effectively blocking cancer cell proliferation. Curcumin commonly induces cell cycle arrest at various checkpoints, predominantly at the G2/M and S phases, depending on the cancer cell type and context. For instance, in hepatocellular carcinoma cells, curcumin treatment results in S-phase arrest accompanied by decreased expression of cyclin B1 and CDK1, essential regulators of cell cycle progression. Similarly, curcumin analogs induce G2/M phase arrest in glioma and osteosarcoma cells by modulating cyclin and CDK expression, thus halting mitotic progression. The modulation of cell cycle regulatory proteins extends to the suppression of oncogenic signaling pathways, such as Wnt/β-catenin, which controls the expression of cyclin D1 and c-myc, further contributing to cell cycle blockade. Moreover, curcumin influences the expression of tumor suppressor genes, including p21 and p53, which enforce cell cycle checkpoints and facilitate apoptosis.

The synergistic integration of apoptosis induction and cell cycle arrest by curcumin is enhanced when combined with other therapeutic agents. For example, co-treatment with curcumin and chemotherapeutic drugs, such as docetaxel, melphalan, or tamoxifen, potentiates apoptosis and cell cycle arrest, thereby improving anticancer efficacy in prostate, breast, and other cancer models. Additionally, curcumin analogs and hybrid molecules have been designed to increase structural stability and bioavailability, demonstrating an enhanced ability to induce G2/M arrest and apoptosis via pathways such as JNK/c-Jun signaling. The modulation of apoptotic and cell cycle proteins by curcumin also involves epigenetic mechanisms, including the inhibition of DNA methyltransferase 1 (*DNMT1*), leading to the reactivation of tumor suppressor genes and apoptosis-related genes.

Collectively, these findings underscore the multifaceted role of curcumin in regulating apoptosis and cell cycle progression through mitochondrial and death receptor pathways, modulation of key regulatory proteins (e.g., Bax, Bcl-2, caspases, cyclins, and CDKs), and interference with oncogenic signaling cascades. This dual action not only halts cancer cell proliferation but also promotes programmed cell death, positioning curcumin as a promising functional food component and adjuvant in cancer therapy. Future clinical investigations are warranted to optimize curcumin formulations and combinatorial regimens to fully harness its therapeutic potential.

### 6.4. Immunomodulatory Effects

Curcumin, the principal bioactive compound derived from turmeric (*Curcuma longa*), exhibits profound immunomodulatory effects on various immune cells, notably, macrophages and T lymphocytes. Studies have demonstrated that curcumin can modulate macrophage function by enhancing their antimicrobial activity, promoting phagocytosis, and regulating the secretion of inflammatory mediators. For instance, *Curcuma longa* extracts have been shown to cooperate with murine macrophages (RAW 264.7) to control opportunistic pathogens by reducing microbial load and modulating inflammation-related molecules, such as IL-1β, TNF-α, IL-10, and nitric oxide, without compromising macrophage viability [52]. Curcumin also influences dendritic cells (DCs), the key antigen-presenting cells bridging innate and adaptive immunity. It induces a tolerogenic phenotype in DCs by inhibiting maturation markers, cytokine production, and antigen presentation capacity, thereby dampening excessive immune activation and promoting regulatory T cell induction. Regarding T cells, curcumin modulates the balance among T helper subsets, including Th1, Th2, Th17, and regulatory T cells (Tregs). It inhibits the proliferation and cytokine production of pro-inflammatory Th17 cells, reducing the levels of IL-17, IL-22, and TNF-α, which are implicated in autoimmune and inflammatory diseases. Additionally, curcumin enhances natural killer (NK) cell-mediated interferon-gamma (IFN-γ) secretion indirectly via macrophage-derived cytokines, such as MIP-1α, CXCL-1, IL-1β, and PAI-1, highlighting its role in orchestrating innate and adaptive immune responses. The immunomodulatory effects extend to B lymphocytes as well, where curcumin influences proliferation, differentiation, and function, including in pathological states, such as B cell lymphoma, although evidence remains limited. Collectively, these findings underscore curcumin’s capacity to fine-tune immune cell functions, balancing activation and tolerance, which is critical for maintaining immune homeostasis and combating diseases.

The immunomodulatory properties of curcumin contribute significantly to its anticancer potential by remodeling the tumor immune microenvironment. Curcumin exerts antitumor effects through multiple mechanisms, including inhibition of tumor cell proliferation, induction of apoptosis, and modulation of immune responses within the tumor milieu [53]. It enhances cytotoxic T lymphocyte (CTL) infiltration and activity, which is correlated with a reduced risk of cancer recurrence, as observed in ovarian and breast cancers. Curcumin also suppresses immunosuppressive cells, such as regulatory T cells and tumor-associated macrophages, thereby reversing immune evasion and promoting anti-tumor immunity. Its ability to inhibit key signaling pathways like NF-κB, STAT3, and MAPKs reduces the production of pro-inflammatory cytokines and chemokines that otherwise foster a tumor-promoting microenvironment. Moreover, curcumin’s anti-inflammatory and antioxidant properties mitigate oxidative stress, further supporting immune cell function and tumor suppression. Innovative delivery systems, such as curcumin-loaded nanoparticles and fibrin matrices, have been developed to enhance bioavailability and sustain immunomodulatory effects, resulting in improved tumor control and reduced metastasis in preclinical models. In combination with radiotherapy, curcumin acts as an immune booster, augmenting abscopal effects by increasing proapoptotic and proinflammatory protein expression and enhancing T cell activation. These multifaceted immunomodulatory actions of curcumin collectively improve the immune microenvironment, transforming immunologically cold tumors into responsive ones, thereby potentiating anticancer therapies.

### 6.5. Preclinical and Clinical Research Progress

Curcumin, the principal bioactive compound in turmeric, has been extensively studied in preclinical models for its anticancer properties, demonstrating multifaceted mechanisms, including anti-inflammatory, antioxidant, antiproliferative, pro-apoptotic, and anti-metastatic effects. In vitro studies reveal that curcumin inhibits the proliferation of various cancer cell lines such as breast, colorectal, pancreatic, liver, glioma, and ovarian cancers by modulating key signaling pathways including NF-κB, PI3K/Akt, Wnt/β-catenin, and p53/Bcl-2 axis [54,55]. These pathways regulate cell survival, apoptosis, inflammation, and metastasis, suggesting curcumin’s potential as a chemopreventive and therapeutic agent. For instance, curcumin induces apoptosis and G1-phase cell cycle arrest in hepatocellular carcinoma cells via the EGFR/p53/Bcl-2 signaling axis and inhibits tumor growth and metastasis in breast cancer models, especially when formulated as nanoparticles to enhance bioavailability [56]. Nanoparticle and liposomal formulations have been shown to improve curcumin’s stability, solubility, and targeted delivery, thereby enhancing anticancer efficacy. Additionally, curcumin exhibits synergistic effects with standard chemotherapeutic agents, such as proteasome inhibitors in multiple myeloma and gemcitabine in pancreatic cancer, by modulating apoptosis and cell cycle pathways. Preclinical animal studies have demonstrated curcumin’s ability to reduce tumor volume, inhibit angiogenesis, and suppress cancer stemness markers, indicating its potential to target cancer at multiple levels [55]. However, despite promising preclinical data, curcumin’s poor oral bioavailability and rapid metabolism pose challenges for clinical translation, prompting the development of bioavailability-enhanced formulations, such as nano-curcumin and co-administration with piperine [57]. Moreover, curcumin’s classification as a pan-assay interference compound (PAINS) necessitates cautious interpretation of in vitro results and underscores the importance of rigorous pharmacokinetic and pharmacodynamic studies to establish its in vivo efficacy. Collectively, preclinical evidence supports curcumin’s anticancer potential through modulation of multiple molecular targets and pathways, with advanced delivery systems improving its therapeutic index.

Clinical trials investigating curcumin and turmeric formulations have spanned a broad spectrum of diseases, including cancer, inflammatory disorders, metabolic diseases, and neuropsychiatric conditions, reflecting its pleiotropic pharmacological effects. In oncology, clinical studies have primarily focused on safety, biomarker modulation, and symptom management rather than definitive survival benefits. For example, trials in patients with colorectal cancer have confirmed curcumin’s safety and modulation of inflammatory and oncogenic biomarkers but have not demonstrated clear improvements in progression-free or overall survival [54]. Similarly, clinical trials in breast cancer and pancreatic cancer have explored curcumin as an adjunct to chemotherapy, showing improved tolerability and potential enhancement of therapeutic efficacy, although large-scale randomized controlled trials (RCTs) remain limited [55]. Curcumin’s clinical efficacy in inflammatory diseases, such as osteoarthritis, inflammatory bowel disease (IBD), psoriasis, and periodontitis, has been evaluated in multiple RCTs with generally positive outcomes. Meta-analyses indicate that curcumin reduces pain and inflammation in knee osteoarthritis comparably to standard drugs like paracetamol, with fewer adverse effects [26,58]. In IBD, curcumin supplementation improves clinical remission rates in ulcerative colitis, although evidence in Crohn’s disease is inconclusive. Dermatological applications demonstrate curcumin’s anti-inflammatory and wound-healing properties, with clinical trials reporting improvements in conditions, such as oral lichen planus, denture stomatitis, and psoriasis. However, challenges persist in clinical trial design, including heterogeneity in curcumin formulations, dosages, treatment durations, and outcome measures, which complicate data synthesis and interpretation [59]. Moreover, curcumin’s poor bioavailability limits systemic exposure, necessitating the use of bioavailability-enhanced preparations (e.g., nanoformulations, piperine co-supplementation) that have shown improved clinical efficacy in some trials [60]. Safety profiles in clinical studies are generally favorable, with mild adverse events reported; however, rare cases of turmeric-induced liver injury highlight the need for vigilance. Regulatory challenges also arise because turmeric supplements are classified as dietary products rather than drugs, impacting trial design and claims of efficacy. Overall, these preclinical and clinical findings highlight the potential of curcumin as a promising adjuvant agent for cancer management [33,53,54]. However, itis critical to clarify that current clinical investigations of curcumin in oncology have not demonstrated clear or consistent survival benefits in cancer patients. Most clinical trials are limited by small sample sizes, short follow-up durations, and focus on safety profiles, biomarker modulation, or symptomatic improvement rather than definitive endpoints such as overall survival (OS) or progression-free survival (PFS) [53,55]. Further large-scale, long-term randomized controlled trials (RCTs) are, therefore, warranted to validate the clinical value of curcumin in extending patient survival [53,55,58].

## 7. Pharmacokinetics and Clinical Applications of Curcumin

### 7.1. Absorption, Distribution, Metabolism, and Excretion (ADME)

Curcumin, the principal bioactive polyphenol derived from turmeric (*Curcuma longa*), has been extensively studied for its diverse pharmacological activities, including anti-inflammatory, antioxidant, anticancer, antimicrobial, and metabolic regulatory effects. Despite these promising properties, curcumin’s clinical and therapeutic applications are severely limited by its low systemic bioavailability following oral administration. This low bioavailability primarily stems from multiple intrinsic pharmacokinetic challenges: poor aqueous solubility, limited gastrointestinal absorption, rapid metabolism, and swift systemic elimination. Curcumin’s hydrophobic nature results in poor water solubility, which restricts its dissolution in the gastrointestinal tract, thereby limiting its absorption into systemic circulation. Moreover, once absorbed, curcumin undergoes extensive first-pass metabolism in the liver and intestinal mucosa, involving phase I and phase II detoxification enzymes such as cytochrome P450 isoforms and UDP-glucuronosyltransferases. This leads to rapid conjugation and formation of water-soluble metabolites, which are quickly excreted, further reducing the active curcumin available for therapeutic action [28]. Additionally, curcumin exhibits chemical instability, being photosensitive and prone to degradation under physiological pH and during manufacturing and storage, which further compromises its effective concentration [45]. The combined effect of low solubility, poor absorption, rapid metabolism, and elimination results in curcumin’s low plasma concentrations and short half-life, limiting its pharmacological efficacy in vivo. Several pharmacokinetic studies in animal models have quantified this limitation, with oral bioavailability of curcumin reported to be as low as 5.1%. Most unabsorbed curcumin is excreted in feces, while a considerable portion is metabolized by enteric bacteria in the gut microbiome. Gut microbiota mediate reduction, demethylation, and hydroxylation reactions, generating active metabolites such as tetrahydrocurcumin and dihydrocurcumin, which may partially contribute to the in vivo activities of curcumin, with rapid clearance from systemic circulation [46]. Strategies to overcome these limitations have included co-administration with bioenhancers such as piperine, which inhibits metabolic enzymes and increases absorption, thereby prolonging curcumin’s half-life and enhancing plasma levels. Furthermore, encapsulation in various nanocarriers such as liposomes, niosomes, solid lipid nanoparticles, and nanoemulsions has demonstrated significant improvements in solubility, stability, and absorption, resulting in enhanced bioavailability by several fold compared to free curcumin [45]. Despite these advances, the inherently poor pharmacokinetic profile of curcumin remains a major barrier to its clinical translation, necessitating ongoing research into novel formulations and structural analogues to improve its ADME characteristics [61]. Curcumin undergoes extensive biotransformation primarily in the liver and intestinal mucosa, resulting in the formation of various metabolites that contribute to or modulate its biological activities. Phase I metabolism involves reduction reactions yielding tetrahydrocurcumin, hexahydrocurcumin, and octahydrocurcumin, which retain significant antioxidant and anti-inflammatory properties, sometimes surpassing those of the parent compound [61]. Phase II metabolism predominantly involves conjugation reactions such as glucuronidation and sulfation, producing curcumin glucuronides and sulfates, which are more water-soluble and readily excreted but generally exhibit reduced bioactivity compared to free curcumin [28]. Notably, tetrahydrocurcumin, a major reduced metabolite, has demonstrated potent antioxidant, anti-inflammatory, and anticancer effects in various in vitro and in vivo models, suggesting that curcumin’s therapeutic efficacy may be partly mediated through its metabolites [61]. Additionally, synthetic and natural analogues such as dimethoxycurcumin have been developed to improve metabolic stability and bioavailability while maintaining or enhancing pharmacological potency. The biotransformation of curcumin also influences its interaction with drug-metabolizing enzymes and transporters, which can modulate the pharmacokinetics of concomitant medications, as observed in interactions with immunosuppressants like tacrolimus. Recent in silico and molecular docking studies have elucidated the binding affinities and inhibitory potentials of curcumin and its metabolites on various molecular targets, including enzymes involved in inflammation, cancer progression, and microbial biofilms, highlighting the multifaceted bioactivities of curcumin metabolites. Moreover, nanoformulations encapsulating curcumin have been shown to protect the compound from rapid metabolism, thereby enhancing the systemic availability of both curcumin and its active metabolites. Overall, the metabolic profile of curcumin is complex, with metabolites contributing variably to its pharmacological effects. Understanding these metabolic pathways and the bioactivities of metabolites is crucial for optimizing curcumin-based therapeutics and designing derivatives with improved efficacy and pharmacokinetic profiles.

### 7.2. Strategies to Enhance Bioavailability

The poor bioavailability of curcumin, the principal bioactive compound in turmeric, is a major limitation for its therapeutic application. This is primarily due to its low water solubility, rapid metabolism, and poor gastrointestinal stability. Various carrier systems, including liposomes, nanoparticles, nanoemulsions, and polymeric nanocarriers, have been developed to overcome these challenges, showing promising results in enhancing curcumin bioavailability and clinical potential. Liposomes, spherical vesicles composed of phospholipid bilayers, have been widely studied because they can encapsulate hydrophobic compounds, such as curcumin, improving solubility and protecting them from degradation. For instance, curcumin-loaded bilayer nanoliposomes coated with chitosan and alginate demonstrated a 109-fold increase in plasma bioavailability in rats compared to free curcumin, with controlled release in simulated intestinal conditions, highlighting their potential for oral delivery. Similarly, zein-based nanoparticles have shown a 9-fold increase in oral bioavailability in Wistar rats, outperforming other commercial formulations, indicating the advantage of protein-based nanocarriers for curcumin delivery [45]. Polymeric nanoparticles, such as PLGA (poly(lactic-co-glycolic acid)) nanoparticles encapsulating turmeric extract, have also been developed, exhibiting high encapsulation efficiency (~72%), stability over one month, and controlled curcumin release governed by polymer diffusion and relaxation, although their antioxidant activity was somewhat lower than that of free turmeric extract, suggesting a balance between stability and bioactivity.

Nanoemulsions and self-emulsifying drug delivery systems (SEDS) represent another class of carriers that improve the solubility and absorption of curcumin. For example, turmeric nanoparticles formulated via a sustainable raw-to-nano strategy and incorporated into nanoemulsions significantly enhanced the gastrointestinal stability and bioaccessibility of curcumin, reaching a bioaccessibility of 82.5%, compared to only 9% from curcumin crystals [38]. Surfactin-based nanoencapsulation of curcumin improved bioaccessibility to 40.9% and enhanced cellular uptake and antioxidant activity in Caco-2 cells, demonstrating the efficacy of biosurfactants in nanoformulations [44]. Furthermore, quaternized chitosan-coated nanoemulsions showed superior stability and bioavailability compared to uncoated nanoemulsions, along with enhanced antimicrobial activity, indicating the multifunctional benefits of such coatings.

Protein-based carriers have also been explored for their emulsifying and protective properties. Lupin protein isolate-based nanoparticles protected curcumin from degradation better than oil-in-water emulsions, with 70% recovery of curcumin post-digestion; although transport across cell monolayers remained limited, this highlights the need for further optimization. Whey protein isolate complexes with polysaccharides improved curcumin entrapment, antioxidant activity, and stability against UV and thermal degradation, suggesting that protein-polysaccharide complexes can effectively enhance the functional properties of curcumin.

Nanogel systems have emerged as another promising strategy. A modified nanogel composed of PNIPAM and β-cyclodextrin grafted onto hyaluronic acid achieved high curcumin encapsulation efficiency (~93%) and demonstrated biocompatibility with fibroblast cells, indicating its potential for wound healing applications. Hydrogels, owing to their biocompatibility and low toxicity, have been reviewed as next-generation curcumin delivery systems with applications in anti-cancer and wound healing therapies.

The clinical translation of these carrier systems is supported by pharmacokinetic studies demonstrating significantly enhanced plasma concentrations and area under the curve (AUC) values. For example, a dispersible oleoresin-based turmeric formulation showed a 16.1-fold increase in maximum concentration (Cmax) and a 39-fold increase in AUC of free curcumin compared to standard extracts, confirming the clinical relevance of such formulations [62]. Similarly, a novel solvent-free co-grinding preparation improved curcumin bioavailability by 178-fold over standard curcumin in healthy volunteers, highlighting the potential for scalable, safe, and effective curcumin delivery systems.

In addition to enhancing bioavailability, these carrier systems can improve curcumin stability against environmental factors, such as pH, temperature, and light. For instance, spray-dried curcumin-lecithin complexes with maltodextrin carriers exhibited improved solubility, thermal stability, and protection against degradation in simulated gastrointestinal fluids, supporting their use in functional foods and pharmaceuticals.

Topical and targeted delivery systems have also been explored beyond oral delivery. Mucoadhesive capsules based on bacterial nanocellulose and chitosan have been developed for the gastric delivery of turmeric extracts, demonstrating significant mucoadhesive properties and potential for treating gastric diseases. Niosomes, non-ionic surfactant vesicles, have been investigated for curcumin encapsulation in cancer treatment, offering improved physical and pharmacokinetic properties, controlled release, and enhanced cytotoxicity against malignant cells.

Overall, the development of diverse carrier systems, such as liposomes, nanoparticles, nanoemulsions, protein complexes, nanogels, and novel formulations, has substantially enhanced the bioavailability, stability, and therapeutic efficacy of curcumin. These advances not only improve curcumin’s clinical potential but also expand its applications in functional foods, nutraceuticals, and pharmaceuticals. Future clinical trials and regulatory considerations will be crucial for translating these promising delivery systems into routine clinical and commercial use.

Combination therapy involving curcumin and bioavailability enhancers or other bioactive compounds has been extensively studied to improve curcumin absorption and therapeutic efficacy. Piperine, a major alkaloid in black pepper, is the most widely studied bioavailability enhancer that inhibits hepatic and intestinal glucuronidation, thereby increasing curcumin bioavailability. A randomized, double-blind clinical trial in hemodialysis patients demonstrated that turmeric supplementation combined with piperine significantly reduced oxidative stress markers, such as malondialdehyde and ferritin levels, compared to turmeric alone, indicating improved systemic bioavailability and biological effects [60]. However, caution is warranted, as enhanced bioavailability formulations containing piperine have been linked to rare cases of drug-induced liver injury, underscoring the need for careful dosing and monitoring.

Other combination strategies involve the co-administration of natural compounds or the formulation of food matrices to improve curcumin absorption. For example, turmeric nanoparticles incorporated into nanoemulsions with lipid phases improved gastrointestinal stability and bioaccessibility by facilitating curcumin encapsulation within mixed micelles, enhancing absorption [38]. The use of natural sweeteners, such as mogroside V, to form solid dispersions with curcumin increased its water solubility by nearly 6000-fold and oral absorption by 29-fold in rats, illustrating the potential of combining curcumin with solubilizing agents.

Protein and polysaccharide co-formulations also modulate curcumin absorption. Studies have shown that proteins can act as emulsifiers during digestion, facilitating the transition of lipophilic compounds, such as curcumin, into mixed micelles for absorption. Conversely, poorly digested proteins may impede this process. Polysaccharides, such as carrageenan, have been used to form nanocomposites with curcumin, enhancing intestinal permeability by interacting with specific transporters (e.g., SLC26A2), resulting in a 44-fold increase in permeability compared to free curcumin. Similarly, whey protein isolate combined with chitosan improved curcumin entrapment and antioxidant stability, suggesting synergistic effects of biopolymer complexes on curcumin bioavailability.

The combination of other bioactive phytochemicals can also influence curcumin absorption and efficacy. A study investigating the effects of turmeric, red pepper, and black pepper on carotenoid bioaccessibility found that capsaicin (from red pepper) and piperine (from black pepper) enhanced cellular uptake and transport of carotenoids, whereas curcumin inhibited absorption, indicating complex interactions between these compounds at the enterocyte level. This highlights the importance of understanding synergistic and antagonistic effects in multi-component dietary interventions.

Nanoparticle formulations combining curcumin with other bioactive oils, such as fish oil (docosahexaenoic acid), have demonstrated improved pharmacokinetics, with enhanced absorption and a shorter time to peak concentration, compared to standard curcumin extracts, suggesting that co-formulation with lipids can potentiate curcumin bioavailability. Additionally, co-encapsulation of curcumin with turmeric essential oil increased free curcumin levels in plasma and brain tissue in animal models, resulting in significant neuroprotective effects, further supporting combination strategies to improve tissue distribution and efficacy.

Moreover, the use of *N*-acetylcysteine (NAC) conjugated bovine serum albumin nanoparticles enhanced intestinal absorption and oral bioavailability of curcumin by 3.25- to 4.42-fold in rats, demonstrating that chemical modification and co-delivery with absorption enhancers can significantly improve curcumin pharmacokinetics.

In summary, combination therapies involving curcumin with bioenhancers such as piperine, co-formulation with lipids, proteins, polysaccharides, or other phytochemicals, and incorporation into advanced nanocarriers have been shown to markedly enhance curcumin absorption and bioavailability. These strategies improve systemic exposure and potentiate curcumin’s therapeutic effects in various disease models. However, safety considerations, especially regarding hepatotoxicity with certain bioenhancers, warrant careful clinical evaluation. Future research should focus on optimizing these combinations to maximize efficacy while minimizing adverse effects and facilitating the translation of curcumin into effective clinical interventions.

### 7.3. Current Clinical Applications and Safety Evaluation

Turmeric (*Curcuma longa*) and its primary bioactive compound, curcumin, have been extensively studied for their therapeutic potential across a broad spectrum of diseases, reflecting their historical use in traditional medicine and recent scientific interest. Clinical trials and systematic reviews have demonstrated turmeric’s efficacy in inflammatory, metabolic, musculoskeletal, dermatologic, infectious, and oncologic conditions. For example, in musculoskeletal health, randomized controlled trials (RCTs) have shown that bioavailable turmeric extracts significantly alleviate the symptoms of knee osteoarthritis, reducing pain, stiffness, and inflammatory biomarkers, such as *C*-reactive protein (CRP) and tumor necrosis factor-alpha (TNF-α) [58,63]. In dermatology, curcumin has been evaluated in RCTs for psoriasis, radiation dermatitis, oral lichen planus, and other skin conditions, with evidence supporting its efficacy and excellent safety profile. Turmeric’s anti-inflammatory and antioxidant properties have also been leveraged in the management of inflammatory bowel disease (IBD), where meta-analyses of placebo-controlled RCTs indicate curcumin’s potential to induce clinical remission in patients with ulcerative colitis. In addition, curcumin has shown notable antibacterial activity against *Helicobacter pylori*, a key pathogen associated with chronic gastritis, peptic ulcer, and gastric cancer. In vitro and in vivo studies have verified that curcumin can inhibit the growth, adhesion, and colonization of *H. pylori* in the gastric mucosa. In regions where *H. pylori* infection is endemic, regular dietary intake of turmeric has been suggested to exert a protective effect by alleviating gastric inflammation and reducing the risk of *H. pylori*-related gastric mucosal lesions [64]. Moreover, curcumin supplementation has shown benefits in glycemic control and insulin resistance in type 2 diabetes mellitus (T2DM), with moderate to high certainty evidence supporting reductions in HbA1c and improvements in metabolic parameters. In oncology, curcumin has been investigated as an adjunct to chemotherapy and radiotherapy, demonstrating the potential to enhance therapeutic efficacy and mitigate adverse effects in cancers, such as breast, colorectal, and acute myeloid leukemia, although clinical evidence remains preliminary and sometimes inconclusive [65]. Additionally, turmeric and curcumin have been studied in infectious diseases, including COVID-19, where meta-analyses of RCTs suggest that bioavailability-enhanced formulations, such as nano-curcumin, reduce mortality and improve clinical outcomes when used adjunctively [66]. Other emerging applications include the management of polycystic ovary syndrome (PCOS), rheumatoid arthritis, diabetic retinopathy, and hepatobiliary diseases, where curcumin’s anti-inflammatory, antioxidant, and immunomodulatory effects contribute to clinical improvements [47]. Collectively, these clinical studies underscore turmeric’s broad therapeutic potential, with many trials employing advanced formulations to overcome curcumin’s inherent low bioavailability, thereby enhancing clinical efficacy.

The safety profile of turmeric and its extracts, particularly curcumin, has been extensively evaluated in clinical settings, generally demonstrating good tolerability and a low incidence of adverse effects. Multiple clinical trials have reported that turmeric supplementation, even at relatively high doses or over extended periods, does not significantly alter liver function tests or hematological parameters or induce serious toxicity in healthy individuals or patients with chronic diseases [67]. For instance, a prospective clinical study assessing a standardized turmeric extract (PUREMERIC™) in healthy participants found no significant changes in liver enzymes or hematological indices after 90 days of supplementation, contradicting earlier case reports of turmeric-induced liver injury [67]. In patients with osteoarthritis, turmeric extracts were associated with fewer and milder adverse events compared to conventional medications, such as paracetamol, highlighting a favorable safety and tolerability profile [58]. Dermatological trials similarly confirm curcumin’s excellent safety, with only mild and infrequent adverse events reported. However, isolated case reports and pharmacovigilance data caution about the potential for drug-induced liver injury (DILI) associated with turmeric-containing dietary supplements, especially when combined with bioavailability enhancers, such as black pepper (piperine), underscoring the need for careful patient monitoring and disclosure of supplement use. Another concern regarding the safety of turmeric involves the potential accumulation of toxic metals, particularly arsenic (As), when plants are cultivated in volcanic soils. Volcanic regions are known for high mineral content, raising theoretical concerns about food safety. However, a recent field study directly addressing this issue reported that turmeric grown on volcanic rocks in Central Java, Indonesia, contained arsenic levels as low as 0.005–0.400 mg/kg, which is considered very low and below safety thresholds of concern [68]. The study also noted that while elements like aluminum and iron were elevated, these were primarily non-toxic nutrients derived from the volcanic substrate [68]. Thus, current evidence does not support the hypothesis that volcanic soils universally lead to toxic arsenic accumulation in turmeric. Furthermore, turmeric and curcumin exhibit interactions with drug-metabolizing enzymes such as carboxylesterase 1 (CES1), which may influence the pharmacokinetics of co-administered medications, suggesting the possibility of botanical-drug interactions that require further clinical investigation. In pediatric populations, curcumin supplementation has been well tolerated at various doses and durations, with no serious adverse effects reported, although larger controlled trials are needed to confirm long-term safety. Overall, while turmeric and curcumin are generally safe and well-tolerated, attention to formulation, dosage, patient comorbidities, and potential herb-drug interactions is essential to minimize risks. The development of novel formulations, such as nanocurcumin and water-dispersible extracts, aims to optimize therapeutic outcomes while maintaining safety. Continued rigorous clinical trials and pharmacovigilance are warranted to further delineate safety profiles across diverse patient populations and clinical indications.

In terms of safety profile, curcumin is generally well tolerated in clinical trials, with most adverse effects being mild and transient, including gastrointestinal discomfort, nausea, and diarrhea. However, potential drug–drug interactions should be carefully considered, particularly with anticoagulants, antiplatelet agents, and metabolizing enzyme substrates, as curcumin may modulate cytochrome P450 enzyme activities. Notably, while hepatotoxicity is rarely reported at standard doses, isolated case studies and preclinical evidence have raised concerns about liver injury at high doses or in susceptible individuals, warranting careful monitoring of liver function in long-term or high-dose administration. Further pharmacovigilance studies are needed to fully characterize its safety profile in vulnerable populations [69,70,71].

### 7.4. Future Directions for Clinical Research

Curcumin, the principal bioactive compound of turmeric, demonstrates a broad spectrum of pharmacological activities, including anti-inflammatory, antioxidant, anticancer, and neuroprotective effects, which are increasingly attributed to its ability to modulate epigenetic mechanisms. Epigenetic regulation involves reversible modifications, such as DNA methylation, histone modification, and non-coding RNA expression, that influence gene expression without altering the DNA sequence. Preclinical studies have shown that curcumin can influence these epigenetic processes by inhibiting DNA methyltransferases, modulating histone acetyltransferases and deacetylases, and regulating microRNAs, thereby affecting pathways involved in inflammation, cancer progression, and metabolic diseases. Despite these promising mechanistic insights, clinical validation of curcumin’s epigenetic effects remains limited. Current clinical trials predominantly focus on curcumin’s anti-inflammatory and antioxidant outcomes in conditions, such as osteoarthritis, metabolic syndrome, and various cancers, but rarely assess epigenetic biomarkers or gene expression changes in human subjects. Moreover, curcumin’s poor bioavailability has posed challenges in translating epigenetic modulation observed in vitro to clinical settings. Recent advancements in formulation technologies, such as nano-curcumin and cyclodextrin-complexed curcumin, have improved systemic absorption and stability, offering opportunities to better evaluate epigenetic impacts in vivo. Future clinical research should incorporate well-designed randomized controlled trials that include epigenetic endpoints, such as DNA methylation profiles, histone modification patterns, and miRNA expression, in target tissues or accessible surrogate samples. Such studies would clarify the extent to which curcumin’s therapeutic effects are mediated via epigenetic pathways in humans. Additionally, longitudinal studies could elucidate the persistence and reversibility of epigenetic changes induced by curcumin supplementation. Integrating multi-omics approaches and advanced bioinformatics will further enhance our understanding of curcumin’s epigenetic regulatory networks. Ultimately, clinical validation of curcumin’s epigenetic modulation will support its development as a precision therapeutic agent, enabling tailored interventions based on individual epigenetic landscapes and disease states [26,72].

The evolving fields of personalized nutrition and precision medicine aim to customize dietary and therapeutic strategies based on individual genetic, epigenetic, metabolic, and microbiome profiles to optimize health outcomes. Turmeric and its active constituent, curcumin, possess multifaceted bioactivities that align well with these approaches. Clinical studies have demonstrated curcumin’s efficacy in managing musculoskeletal disorders, metabolic syndrome, inflammatory bowel disease, and neurodegenerative conditions, with variable responses observed among individuals. Such inter-individual variability underscores the potential for personalized applications. Advances in nutrigenomics and metabolomics have enabled the identification of genetic polymorphisms and metabolic phenotypes that influence curcumin metabolism, bioavailability, and therapeutic response. For example, variations in genes encoding enzymes involved in curcumin biotransformation or inflammatory mediators may determine efficacy and safety profiles. Furthermore, curcumin’s modulation of the gut microbiota, which itself exhibits considerable inter-personal diversity, suggests an avenue for tailoring interventions to optimize microbiome-mediated effects on systemic inflammation and metabolism. The integration of curcumin supplementation with individual molecular and clinical data can facilitate precision dosing, formulation selection (e.g., nanoformulations for enhanced bioavailability), and combination therapies to maximize benefits while minimizing adverse effects. Additionally, emerging technologies, such as artificial intelligence and machine learning, can analyze complex datasets to predict responders and non-responders, guiding clinical decision-making. Future clinical research should focus on stratified trials that incorporate genotyping, epigenetic profiling, and microbiome analysis to establish biomarkers predictive of curcumin responsiveness. Such studies will inform guidelines for personalized turmeric-based interventions in preventive and therapeutic contexts. Moreover, the development of functional foods and nutraceuticals enriched with bioavailable curcumin tailored to individual nutritional needs represents a promising direction. By harnessing the synergy of turmeric’s bioactive compounds with personalized medicine frameworks, it is feasible to advance curcumin from a broadly used supplement to a targeted, evidence-based modality within integrative healthcare [31,72].

## 8. Conclusions

In conclusion, the evolving landscape of turmeric research underscores its significant potential as a functional food with profound implications for cancer prevention and therapy. From an expert perspective, turmeric, particularly its principal bioactive compound, curcumin, embodies a complex phytochemical profile that exerts multifaceted biological activities. These activities, including antioxidant, anti-inflammatory, pro-apoptotic effects, and modulation of epigenetic mechanisms such as non-coding RNA networks, collectively contribute to its promising anticancer properties. The integration of these diverse molecular pathways highlights the intricate balance that turmeric maintains in targeting cancer pathophysiology, positioning it as a unique candidate in the field of complementary and integrative oncology.

However, it is necessary to acknowledge several critical limitations in the current literature. Potential publication bias may exist, with a tendency to emphasize positive findings while underreporting inconclusive or negative results. Moreover, despite extensive research efforts devoted to turmeric and curcumin, the pragmatic challenge of low bioavailability remains largely unresolved, restricting its clinical translational potential.

Although curcumin’s pharmacokinetic properties are less favorable compared with some other chemopreventive agents, its remarkable safety, long dietary history, pleiotropic biological activities, and capacity for long-term intervention still support the value of continued research. Rather than conducting excessively extensive studies, future investigations should prioritize optimized delivery systems, rigorous clinical trial designs, clear clinical endpoints, and comparative evaluations with more pharmacokinetically favorable agents. Such a focused strategy will help generate more reliable evidence and practical guidelines for the development of turmeric-based functional foods and chemopreventive interventions.

The journey from laboratory findings to clinical applications is further challenged by the pharmacokinetic limitations inherent to curcumin, which have prompted extensive research into novel delivery systems and formulation strategies. These advances are critical for translating promising in vitro and in vivo results into clinically meaningful outcomes. While current clinical evidence is encouraging, it remains preliminary and highlights the need for large-scale, rigorously designed trials to validate efficacy and clarify safety profiles across diverse populations.

Integrating preclinical evidence with balanced clinical interpretation is essential to bridge mechanistic insights with practical therapeutic value. Future research should continue to explore the precise molecular mechanisms of turmeric and curcumin, while addressing heterogeneity in tumor biology and patient-specific responses. This precision-oriented approach can help optimize therapeutic strategies and maximize the functional potential of turmeric.

Ultimately, turmeric represents a paradigmatic example of a natural product with multifactorial bioactivity and considerable translational promise. Its further development depends on overcoming pharmacokinetic barriers, substantiating clinical efficacy and safety through high-quality evidence, and deepening mechanistic understanding within a precision medicine framework. This balanced, comprehensive approach will determine the future role of turmeric as a functional food and effective chemopreventive agent in modern healthcare.

## Data Availability

No new data were created or analyzed in this study. Data sharing is not applicable to this article.
